# Impact of Dry Eye Syndrome on Vision-Related Quality of Life in a Non-Clinic-Based General Population

**DOI:** 10.1186/1471-2415-12-22

**Published:** 2012-07-16

**Authors:** Qihua Le, Xiaodong Zhou, Ling Ge, Liangcheng Wu, Jiaxu Hong, Jianjiang Xu

**Affiliations:** 1 Department of Ophthalmology, Eye & ENT Hospital of Fudan University, No.83 Fenyang Road, Xuhui District, Shanghai, 200031, China; 2 Department of Ophthalmology, Jinshan Hospital of Fudan University, No.391 East Jinyi Road, Jinshan District, Shanghai, 200540, China; 3 Department of Ocular Surface Disease, Shanghai Eye Disease Prevention & Treatment Center, No. 380 Kangding Road, Jing’an District, Shanghai, 200040, China; 4 Department of Ophthalmology, Jing’an District Center Hospital, No.259 Xikang Road, Jing’an District, Shanghai, 200040, China

**Keywords:** Dry eye syndrome, NEI VFQ-25, Visual quality of life

## Abstract

**Background:**

Dry eye syndrome (DES) is a common ocular disorder occurring in general population. The purpose of this study is to evaluate the impact of DES on vision-related quality of life (QoL) in a non-clinic-based general population.

**Methods:**

This population-based cross-sectional study enrolled subjects older than 40 years, who took part in an epidemiological study on dry eye in Sanle Community, Shanghai. Apart from the collection of sociodemographics, dry eye symptoms, and other clinical data, a Chinese version of the 25-item National Eye Institute Visual Functioning Questionnaire (NEI VFQ-25) was administered to all subjects. Comparisons of the NEI VFQ-25 subscale item scores and composite score were made among subgroups divided according to the presence of dry eye symptoms or signs. Multivariate regression analysis was performed to investigate the relationship between the clinical variables and the VFQ-25 composite score.

**Results:**

A total of 229 participants were enrolled in the study, with an average age of (60.7 ±10.1) years old. Majority of these participants were female (59.8 %, 137/229). The total DES symptom scores (TDSS) in subjects either with definite DES or only with dry eye symptoms were significantly higher (F = 60.331, P < 0.001). The values of tear break-up time (TBUT) and Schirmer test were significantly lower in participants with DES and those with dry eye signs only (F = 55.158 and 40.778, P < 0.001). The composite score of the NEI VFQ-25 was significantly lower in subjects with DES (F = 4.901, P = 0.003). Moreover, the subscale scores of ocular pain and mental health were significantly lower in those with either DES or dry eye symptoms only (F = 10.962 and 7.362 respectively, both P < 0.001). The multiple regression analysis showed that the TDSS had a significant negative correlation with the VFQ-25 composite score as well as with the subscale score for ocular pain and mental health, even after the adjustment of all other factors (all P < 0.01).

**Conclusions:**

The symptoms of dry eye are associated with an adverse impact on vision-related QoL in non-clinic-based general population, which is mainly represented as more ocular pain and discomfort, and impaired mental health as well. Apart from clinical examination, it is also important to refer to subjective symptoms and QoL scores when assessing the severity of DES.

## Background

Dry eye syndrome (DES) is a multifactorial disease of tear and ocular surface, which can cause symptoms of discomfort, visual disturbance, and tear film instability [1]. The estimated prevalence of DES in Chinese population has been reported to be ranging from 21 % to 50.1 % in adult aged 40 years or older [2-5]. In recent years, DES has been recognized as a growing public health problem and one of the most frequent reasons for seeking eye care.

The medical treatment and clinical studies performed on DES have conventionally focused mainly on physician-based outcomes, such as the improvement of epithelium healing and tear film stability [6,7]. However, recent and improved understanding of the pathogenesis and therapeutic targets of DES has encouraged increased awareness of this disease among patients and clinicians, stimulating increased recognition of DES-associated impairments in quality of life (QoL). QoL broadly encompasses physical, social/role, psychological/emotional, and cognitive functioning concepts. QoL measures patient-reported data that may not be obtained through objective measures. In general, vision-related QoL is an important outcome in the evaluation of therapeutic decisions as well as in the assessment of the impact of any ocular condition on economic and public health.

The 25-item National Eye Institute Visual Function Questionnaire (NEI-VFQ), a method designed for the study of vision-specific QoL, has been employed in several DES research that are mainly focused on outpatients diagnosed with DES [8-10]. Nevertheless, the importance of DES on QoL has been underestimated to some extent because only those with relatively severe DES and seeking doctors’ help have been investigated heretofore. However, DES is a common ocular surface disease. To the best of our knowledge, its impact on QoL in non–clinic-based general population has not been previously reported. In view of this, we performed a population-based research focused on the QoL of subjects with DES and suspected DES in an inhabitant community of Shanghai.

## Methods

### Study population

The present study is a part of the nationwide epidemiological study on dry eye. A population-based survey performed among subjects that are ≥ 18 years old in Sanle Community, Shanghai, China was conducted between March 15, 2010 and June 30, 2010. Sanle community is located in Jing’an district, the central region of Shanghai. It was chosen as the study community on the basis of its metropolitan location, population stability, and support from local government and medical institutions. The study was approved by the ethics committee of Eye, Ear, Nose and Throat Hospital of Fudan University, and conducted according to the tenets of the Declaration of Helsinki.

Among the participants of the epidemiological study, those ≥ 40 years old were enrolled in this study. Subjects that were found to have the following abnormalities were excluded: blood pressure higher than 160/95 mmHg, diabetes mellitus, autoimmune disorder or other systemic disorders, corneal disorders that would probably affect visual acuity, such as corneal leukoma, pterygium and corneal ulcer, intraocular pressure(IOP) higher than 21 mmHg or lower than 9 mmHg, lens opacity greater than grade II according to the Lens Opacity Classification System III (LOCSIII), any abnormalities in the fundus photograph, refractive error more than +/−6D, and history of contact lens wear, allergic conjunctivitis, or ocular surgery. Those who had been diagnosed with DES before and treated with either artificial tear or punctal occlusions were excluded as well. Informed consent was obtained from all enrolled participants.

### Ocular examination

Before ocular examination, trained interviewers contacted the participants and administered a structured questionnaire, which included detailed demographic information (age, gender, educational level, occupation), history of systematic disorders and ocular diseases, life style (computer use and air conditioner), and dry eye symptoms. The questions regarding dry-eye symptoms, which had previously been used by Schein et al. [11], consisted of six items: (1) Do your eyes ever feel dry? (2) Do you ever feel a gritty or sandy sensation in your eyes? (3) Do your eyes ever have a burning sensation? (4) Are your eyes ever red? (5) Do you notice much crusting on your lashes? and (6) Do your eyelids ever get stuck? For each question, the participants were asked to choose among the following answers: never, rarely, sometimes, often, or all the time, which were scored as 0, 1, 2, 3 and 4 accordingly. Total DES symptom score (TDSS) was calculated by the interviewers according to the method reported previously [12].

All subjects underwent a complete ophthalmologic examination, including best-corrected visual acuity, slitlamp biomicroscopy, direct ophthalmoscopy, fundus photography, tonometry, and optometric examination. All pieces of equipment were transported to a local medical unit for the convenience of the participants. The best corrected visual acuity (BCVA) was evaluated with the LogMAR visual acuity chart. Noncontact tonometer rather than Goldmann applanation tonometer was used to measure IOP so as to avoid the impact on corneal epithelium and fluorescence staining.

The dry eye examinations included tear-film breakup time test (TBUT), fluorescein staining score (FSS) of the cornea, Schirmer I test (ST) and slit-lamp assessment of anterior segment. The examination was performed in conformity to the procedures described in previous report [3]. The ophthalmologists performing the eye examinations were blinded to the dry eye information found in the questionnaire.

### Diagnostic criteria of DES and division of participants

Dry eye was defined as the simultaneous presence of significant symptoms and at least one sign. Subjects were considered symptomatic when at least one of the symptoms mentioned in the questionnaire was experienced often or all the time. Objective tests were considered indicative of signs in the following instances: ST score ≤ 5 mm, TBUT ≤ 10 seconds, and FSS ≥ 1. These criteria had to be met in at least one eye. Subjects with definite dry eye were classified as group A. The asymptomatic subjects—those only with signs of DES—were defined as type I suspected DES (group B). Those who reported significant symptoms but had no signs of DES were considered as type II suspected DES (group C). The participants were classified into group D if they had neither dry eye symptoms nor signs.

### Visual Function Questionnaire (VFQ-25)

A Chinese version of the VFQ-25, which was used in the previous research [13,14], was administered to all the enrolled subjects. All subjects were requested to fill up the questionnaire by themselves. The research staff explained the questionnaire to the participants and provided assistance when required. For the illiterate participants, the research staff read the questionnaire for them in a neutral and uniform manner and recorded the patients’ choices. The completed questionnaires were reviewed by the research staff to ensure no data were missing.

NEI VFQ-25 with an additional question, being translated into Chinese, was used in the current study to evaluate the vision-specific QoL. Since the response rate of No.14 item was rather low in Chinese population [13], we chose item A8 from NEI-VFQ39 to serve as the appendix of No.14 item according to the instruction of the NEI-VFQ25 manual. If the statistical analysis showed a low response rate of No. 14 item in the current study, the result of its appendix would be used so as to alleviate the impact of high miss rate of No.14 item on the validity and reliability of the whole questionnaire. Each item in the questionnaire was assigned to one of the 12 subscales: general health, general vision, ocular pain, near activities, distance activities, social functioning, mental health, role difficulties, dependency, driving, color vision and peripheral vision. Answers to each question on the VFQ-25 were converted to a 100-point scale in which 100 represent the best possible score or the minimal subjective impairment, and 0 represent the worst or the maximal. The guidelines published by the NEI were adhered to when calculating the above scale conversions and subscale scores.

### Statistical analysis

The mean scores and standard deviations were calculated for each VFQ-25 subscale and composite score. ANOVA and Chi-square test was used to analyze the age, gender distribution, educational level, occupation and use of computer and air conditioner among the four groups. ANOVA was used to compare each VFQ-25 subscale score and the composite score among the four groups, and Post Hoc Tests were further performed to determine the difference between every two groups. In addition, multivariate regression analysis was performed to investigate the relationship between the clinical variables and the VFQ-25 scores. The covariates tested were age, gender, educational level, occupation, BCVA of the eye with more severe DES, BCVA of the eye with less severe DES or without DES, computer use, use of air conditioner, TDSS, TBUT, ST and FSS. All tests of association were considered statistically significant at P <0.05 (SPSS for Windows, version 13.0; SPSS, Inc., Chicago, IL).

## Results

The number of residents in Sanle Community was 1686, as recorded in the household registration system. A total of 1335 residents took part in the epidemiological study on DES,with a response rate of 79.2 %. The number of those aged 40 years and older was 749. Of this number, 229 were eligible and enrolled in the study of DES on QoL based on the inclusion criteria.

The demographic data of the enrolled participants are listed in Table 1. No significant difference can be found among the four subgroups in terms of average age, occupation, and educational level of participants. However, gender distribution was significantly different among the four subgroups, with group A and C having more female participants (*X*^2^ = 10.823, P = 0.013).

**Table 1:** Demographic data of enrolled participants

**feature**		**total (n = 229)**	**group A (n = 70)**	**group B (n = 50)**	**group C (n = 49)**	**group D (n = 60)**
sex	male	92	26	26	11	29
	female	137	44	24	38	31
age(yrs)	average	60.7 ± 10.1	61.8 ± 9.91	61.6 ± 10.6	59.6 ± 10.9	59.5 ± 9.0
	range	41-86	43-85	41-86	42-83	41-83
occupation	employed	46 (20.1 %)	17	9	9	11
	unemployed	12 (5.2 %)	3	3	4	2
	retired	171 (74.7 %)	50	38	36	47
education	primary school	10 (4.4 %)	3	2	1	4
	junior middle school	15 (6.5 %)	4	4	4	3
	senior middle school	163 (71.2 %)	51	33	34	45
	university	31 (13.5 %)	4	10	9	8
	post-graduate	10 (4.4 %)	8	1	1	0

BCVA, IOP, TDSS, TBUT, ST, and FSS of the four groups are listed in Table 2. Statistically significant differences could be found in neither BCVA nor IOP among the four subgroups. The TDSS of group A and group C were significantly higher than those of group B and group D (F = 60.331, P < 0.001). Moreover, significantly lower values of TBUT were found in group A and group B compared with group C and group D (F = 55.158, P < 0.001). The comparisons on the ST showed similar results (F = 40.778, P < 0.001). No significant difference was found in FSS among the four groups.

**Table 2:** BCVA, IOP, TDSS, TBUT, ST and FSS of four subgroups

	**group A (n = 70)**	**group B (n = 50)**	**group C (n = 49)**	**group D (n = 60)**
BCVA				
right eye	0.23 ± 0.20 (0.16)	0.23 ± 0.22 (0.2)	0.26 ± 0.27 (0.24)	0.25 ± 0.27 (0.2)
left eye	0.27 ± 0.28 (0.2)	0.24 ± 0.22 (0.2)	0.24 ± 0.19 (0.2)	0.24 ± 0.23 (0.2)
IOP				
right eye	15.33 ± 4.61 (15)	15.86 ± 3.99 (15)	16.15 ± 5.51 (16)	16.22 ± 4.49 (16)
left eye	15.21 ± 4.55 (15)	16.17 ± 4.21 (16)	15.52 ± 4.68 (15)	14.93 ± 5.73 (15)
TDSS				
right eye	6.74 ± 4.29 (6)	0.64 ± 1.24 (0)*	5.90 ± 3.81 (6)	0.36 ± 0.73 (0)*
left eye	6.68 ± 4.64 (6)	0.59 ± 1.19 (0)*	5.98 ± 3.46 (6)	0.35 ± 0.79 (0)*
TBUT (s)				
right eye	4.74 ± 3.54 (3)*	4.08 ± 3.29 (3)*	10.25 ± 2.47 (10)	9.98 ± 2.41(9)
left eye	4.33 ± 3.08 (3)*	4.52 ± 3.01 (4)*	10.11 ± 2.60 (10)	10.17 ± 2.22(10)
ST (mm)				
right eye	4.11 ± 3.75(3)*	6.22 ± 4.30 (5)*	11.47 ± 4.27 (10)	12.17 ± 4.52 (12)
left eye	4.20 ± 3.33(3)*	5.94 ± 4.19 (5)*	11.58 ± 5.12 (9)	12.25 ± 4.47 (12)
FSS				
right eye	0.04 ± 0.27 (0)	0	0	0
left eye	0.04 ± 0.27 (0)	0.02 ± 0.14 (0)	0	0

The data were expressed as mean ± SD, with the median in the bracket.

* means P value less than 0.001.

BCVA: Best corrected visual acuity, IOP: intraocular pressure, TDSS: total DES symptom score, TBUT: tear-film breakup time test, ST: Schirmer test, FSS: fluorescein staining score.

The mean scores of each VFQ-25 subscale are all listed in Table 3, ranging from 61.0 for general health to 96.5 for color vision. The mean composite score was 89.4 ± 14.2. Since the missing rate of driving subscale was rather high (219/229, 95.6 %), we omitted this subscale in the calculation of the composite score and further analysis according to the suggestion of previous research [13].

**Table 3:** The mean scores for each subscale and composite score of VFQ-25 in all subjects

**subscale**	**average ± SD**
General Health (n = 229)	60.5 ± 14.1
General Vision (n = 229)	67.8 ± 12.0
Ocular Pain (n = 229)	87.4 ± 17.6
Near activity (n = 229)	85.9 ± 16.2
Distance activity (n = 229)	94.2 ± 12.0
Social function (n = 229)	96.3 ± 11.1
Mental health (n = 229)	87.4 ± 18.2
Role difficulties (n = 229)	88.7 ± 21.5
Dependency (n = 229)	93.3 ± 17.5
Color vision (n = 229)	96.5 ± 11.7
Peripheral vision (n = 229)	95.9 ± 12.3
driving (n = 10)	99.2 ± 2.6
Composite score (n = 229)	89.4 ± 14.2

The composite score was significantly lower in group A, compared with group B and group D (F = 4.901, P = 0.003). The analysis on each subscale score among the four subgroups revealed significant differences in the scores of ocular pain and mental health, which were significantly lower in group A and C, as shown in Table 4 (F = 10.962 and 7.362 respectively, both P < 0.001). The result of multiple regression analysis is listed in Table 5 and it shows that the TDSS has a significant correlation with the VFQ-25 composite score, as well as with the subscale score for ocular pain and mental health, even after the adjustment of other factors (all P < 0.01). Meanwhile, none of the other clinical factors had a significant correlation with the VFQ-25 composite score.

**Table 4:** The analysis of composite score and each subscale score among four subgroups

	**group A (n = 70)**	**group B (n = 50)**	**group C (n = 49)**	**group D (n = 60)**
General Health	57.8 ± 13.5	60.1 ± 12.9	62.9 ± 15.4	62.1 ± 14.6
General Vision	65.5 ± 13.8	70.5 ± 9.6	66.3 ± 11.6	69.5 ± 11.5
Ocular Pain	78.9 ± 21.3 §	92.2 ± 14.4	84.9 ± 14.5†	94.2 ± 13.1
Near activity	81.8 ± 19.0	86.7 ± 13.7	89.6 ± 13.6	86.6 ± 15.9
Distance activity	91.2 ± 15.3	94.3 ± 11.5	95.2 ± 9.8	96.6 ± 8.9
Social function	93.6 ± 15.2	96.6 ± 10.6	98.2 ± 6.3	97.5 ± 8.5
Mental health	80.2 ± 23.4‡	92.3 ± 12.8	85.7 ± 17.4	93.0 ± 12.5
Role difficulties	84.7 ± 22.7	91.2 ± 20.5	85.7 ± 25.1	93.8 ± 16.2
Dependency	90.1 ± 19.3	94.1 ± 16.8	92.9 ± 18.9	96.5 ± 14.1
Color vision	93.8 ± 13.9	96.6 ± 15.0	99.0 ± 5.0	97.5 ± 8.9
Peripheral vision	93.4 ± 17.6	97.1 ± 8.2	96.4 ± 10.2	97.5 ± 8.9
Composite score	85.3 ± 14.5†	91.2 ± 10.6	89.5 ± 8.6	92.3 ± 8.3

† P < 0.05, ‡P < 0.01, § P < 0.001.

**Table 5:** Multivariate regression analysis on the association between total DES symptom score and VFQ-25 composite score, ocular pain subscale score, as well as mental health subscale score

		**VFQ-25 composite score**		**ocular pain subscale score**		**mental health subscale score**	
		**β**	**P value**	**β**	**P value**	**β**	**P value**
Total DES symptom score	Model 1	−0.501	<0.001	−0.836	0.001	−0.903	<0.001
	Model 2	−0.444	0.003	−0.842	0.001	−0.824	0.002
	Model 3	−0.448	0.003	−0.867	<0.001	−0.819	0.002
	Model 4	−0.428	0.008	−0.898	0.001	−0.811	0.003

Values of β are standardized regression coefficients:

Model 1, crude.

Model 2, after the adjustment for age, gender, educational level and occupation.

Model 3, after further adjustment for BCVA of the eye with more severe DES, BCVA of the eye with less severe DES or without DES, use of computer, and use of air conditioner.

Model 4, after further adjustment for TBUT, ST and FFS.

None of the clinical variables adjusted in model 2 to 4 met the 0.0500 significance level for entry into the final model.

## Discussion

The impact of DES on QoL was rather underestimated because it tended not to be a common cause of permanent visual morbidity compared with other ocular diseases, such as cataract, glaucoma and age-related macular degeneration. Consequently, the proportion to seek medical treatment is fairly low in Chinese elderly population, especially those with mild and moderate DES. However, the higher incidence of DES in old people, ever-increasing demands of modern life style such as computer use and air conditioner, and prolonged life expectancy in recent year all highlighted the consequence of DES for vision-related QoL in the general population.

It was the first time to report that the composite score of the participants with DES decreased significantly compared with those without DES or with suspected DES, indicating that DES can produce a significantly negative impact on the overall vision-related QoL in a non-clinic-based population who didn’t seek medical care. Moreover, the current study revealed that the subscale score for ocular pain decreased significantly in the general population with DES or dry eye symptom, which was in agreement with previous studies focusing on outpatients diagnosed with DES. It has been reported that the subscale score for ocular pain ranged from 62.5 to 87.5 in patients with DES, indicating that DES can cause more ocular pain or discomfort [8,15]. It has been considered that the adverse impact on QoL caused by DES, at least partially, resulted from ocular pain, especially in patients with severe dry eye such as in Sjogren’s syndrome [16]. Meanwhile, the application of artificial tears or other treatment may improve the signs, symptoms, and QoL associated with DES [17].

Apart from the decreased scores for ocular pain, lower scores for mental health were also found in the participants with DES, indicating that the disorder caused adverse impact not only on physical health, but also on psychological health. Only a few published studies reported the psychological status of DES subjects, showing that DES subjects were more anxious and depressed compared with those without DES [18-20]. On one hand, it is well known that pain or disabilities caused by chronic disease can induce anxiety and depression [21]. On the other hand, psychosomatic aspects, which include depression, stress, and anxiety, could affect subjective ocular symptoms and pain perception [22], forming a vicious cycle. The fact [18] that subjects with severe dry eye, such as Sjogren’s syndrome, experienced increased clinical anxiety or depression supported the hypothesis that more severe DES symptoms could cause more adverse disturbance on mental health and function.

It’s notable that the symptomatic participants also reported lower scores for ocular pain and mental health, even though they didn’t have any definite signs of DES. Meanwhile, asymptomatic subjects with dry eye signs reported similar VFQ-25 scores as compared with normal controls. It highlighted that the symptoms of DES deviated from signs, and DES symptoms rather than signs had an overwhelming impact on VFQ scores, just as shown by the multiple regression analysis. The unpleasant symptoms of dry eye, such as burning or stinging, ocular grittiness, foreign body sensation, blurred vision, and photophobia, and unsatisfying outcome of palliative treatments could contribute to an impaired QoL. Previous studies revealed the lack of concordance between patient-reported symptoms of DES and clinical parameters (TBUT, ST, and FSS) [23], as well as the absence of correlation between the objective ocular surface examination findings and the VFQ-25 or SF-8 scores [4], which were in agreement with our study. Symptoms, being the most common motivation for seeking eye care, should therefore be a critical outcome measure when assessing treatment effect and improvement of QoL.

Ocular Surface Disease Index (OSDI) [24], a 12-item questionnaire, is a disease-specific measure that explores the vision-related function, ocular symptoms, and environmental triggers related with DES. In contrast, NEI VFQ-25 is a vision-specific (but not disease-specific) method to measure QoL. In addition to an overall composite score ranging from 0 to 100 (lower scores indicating greater impairment), the measure yields general numerous subscales, including general vision, ocular pain, near vision, distance vision, social functioning, mental health, role functioning, dependency, driving, color vision, and peripheral vision [25]. It has been shown that the NEI-VFQ scores were moderately to strongly correlated with scores on the disease-specific OSDI in patients with Sjogren’s syndrome, suggesting the measures were similar in their ability to assess impact of dry eye on vision-targeted QoL [16]. However, the emphasis of these two measures was different to a certain extent. The OSDI is targeted to assess how much the symptoms of dry eye affect the patients’ current status (i.e., in the past week), while the NEI-VFQ may be more suited for capturing the overall impact of a chronic ocular disease on QoL, especially giving consideration to physical health and psychological health simultaneously [14].

In the present study, the diagnosis of DES was done mainly based on both the presence of dry eye symptoms and clinical assessment, which included decreased TBUT, reduced values of Schirmer test and positive corneal staining, according to the criteria of 2007 Dry Eye Work Shop [1]. However, more recently published studies document that an increased tear osmolarity is the hallmark of dry eye disease [26-28]. It has been demonstrated that tear film osmolarity is the single best marker of disease severity across normal, mild/moderate, and severe categories, while other tests, such as Schirmer test without anesthesia, TBUT, corneal staining, meibomian dysfunction assessment, conjunctival staining, and dry eye symptom questionnaire, were found to be informative in the more severe forms of disease [26]. With the consideration that DES severity of the majority subjects in the community population was mild or moderate, tear film osmolarity should have been a better diagnostic tool for differentiating DES and normal controls, as well as for evaluating disease severity. Unfortunately, the device for the clinical testing of tear osmolarity, named as TearLab Osmolarity System [28], had not been available in China when the research was performed. It is urgently required to apply tear osmolarity in further studies involving DES.

Another limitation of the current study is that the status of meibomian glands had not been examined. Therefore, the dry eye symptoms resulting from meibomian gland dysfunction (MGD) cannot be discriminated from those caused by deficient aqueous tear secretion. It has been reported that no correlation has been found between Schirmer result and meibomian gland anomalies [5]. Thus, it’s possible that some symptomatic subjects who have no DES signs can be classified as DES if the assessment of the meibomian glands is carefully performed. However, due to the fact that no significant differences had been found in VFQ score between the participants with DES and those only with DES symptoms, the bias caused by misclassification could be largely neglected. The impact of MGD on vision-related QoL and the differences of QoL between subjects with MGD and other subtypes of DES merit further investigation.

## Conclusions

In conclusion, the result of this population-based study shows that the symptoms of dry eye are associated with an adverse impact on vision-related QoL in non-clinic-based general population, mainly representing as more ocular pain and discomfort, as well as impaired mental health. Examination findings remain essential for the assessment of dry eye, but it is also important to refer to subjective symptoms and QoL scores to supplement the diagnosis and to evaluate treatment effects. Using certain measures to popularize public education on the importance of DES will be helpful in improving the QoL of the general population. The finding adds further insight into the consideration of DES as a significant public health problem that deserves further study.

## Abbreviations

DES, Dry eye syndrome; QoL, quality of life; NEI VFQ-25, 25-item National Eye Institute Visual Functioning Questionnaire; BCVA, corrected visual acuity; IOP, intraocular pressure; TDSS, total DES symptom score; TBUT, tear-film breakup time test; FSS, fluorescein staining score; ST, Schirmer I test; OSDI, Ocular Surface Disease Index.

## Competing interests

The authors declare that they have no competing interests.

## Authors’ contribution

Le Q participated in data collection and drafted the article. Zhou X, Ge L and Wu L participated in data collection and questionnaire administration. Hong J participated in questionnaire administration and gave help in statistical analysis. Xu J played a predominant role in the design and conceptualization of the article. All authors have revised the manuscript critically for important intellectual content and have approved the final manuscript.

## Pre-publication history

The pre-publication history for this paper can be accessed here:

http://www.biomedcentral.com/1471-2415/12/22/prepub
